# III SBC Guidelines on the Analysis and Issuance of Electrocardiographic Reports - Executive Summary

**DOI:** 10.5935/abc.20160173

**Published:** 2016-11

**Authors:** Carlos Alberto Pastore, Nelson Samesima, Horacio Gomes Pereira-Filho

**Affiliations:** Heart Institute (InCor) - Hospital das Clínicas da Faculdade de Medicina, Universidade de São Paulo

**Keywords:** Electrocardiography, Outcome Assessment (Health Care), Guidelines, Abstracts

## Abstract

The third version of the guidelines covers recently described topics, such as ion channel diseases, acute ischemic changes, the electrocardiogram in athletes, and analysis of ventricular repolarization. It sought to revise the criteria for overloads, conduction disorders, and analysis of data for internet transmission.

## Electrocardiographic report^1-4^

### Descriptive report

- analysis of the rhythm and quantification of the heart rate (HR).
- analysis of the duration, amplitude and morphology of the P wave, and duration of the PR interval.
- determination of the electrical axis of P, QRS, and T.
- analysis of the duration, amplitude, and morphology of the QRS complex.
- analysis of ventricular repolarization and description of ST-T, QT, and U changes, when present.

**Conclusive report** - Synthesis of the diagnoses listed in these guidelines.

## Analysis of the cardiac rhythm

**Sinus Rhythm (SR)** - Rhythm observed by the occurrence of positive P waves in the D1, D2, and aVF leads.

**Cardiac Arrhythmia** - Change in frequency, formation, and/or conduction of the electrical impulse across the myocardium.

**Supraventricular arrhythmia -** Rhythm that originates above the junction between the atrioventricular (AV) node and the bundle of His.

**Ventricular arrhythmia -** Rhythm that originates below the bifurcation of the bundle of His, usually visualized as a widened QRS.

**Frequency and Rhythm of the Sinus P Wave** - Normal HR range: 50-100 bpm.^4^

***Sinus bradycardia (SB)*** - Heart rate under 50 bpm.

***Sinus tachycardia*** - Heart rate above 100 bpm.

***Sinus arrhythmia (SA)*** - Usually physiological, depends on the autonomous nervous system and is characterized by a variation in the PP intervals.

### Normal ventricular activation

***Definition of normal QRS*** - Duration < 120 ms and amplitude between 5 and 20 mm (frontal plane) and between 10 and 30 mm (precordial leads), with normal orientation of the electrical axis.^5^

***Normal electrical axis in the frontal plane*** - Normal limits of the electrical axis (frontal plane): between -30° and +90°.

***Normal ventricular activation in the horizontal plane*** - Characteristic: transition from the rS morphology, characteristic of V1, to a typical qR in V6, with r progressively increasing to the maximum in V5.^4^

***Ventricular repolarization*** - This ECG analysis is extremely complex, as it represents the interaction of various systems that are expressed in the segments and in the electrical waves.

***Normal ventricular repolarization*** - Period between the end of the QRS to the end of the T wave or the U wave, when present. Within this period, analyze:

*J point* - End point of the QRS when intersecting with the ST segment.

*ST segment* - Portion of the ECG between the QRS complex and the T wave.

*T wave* - Asymmetric wave with slow onset and fast ending, positive in almost all leads.

*U wave* - Last and smallest deflection in the ECG; when present, it occurs soon after the T wave and before the P wave of the next cycle, with the same polarity as the T wave.

#### QT interval (QT) and corrected QT interval (QTc) ^6^

*QT* - Measurement from the beginning of the QRS to the end of the T wave; represents the total duration of the ventricular electrical activity.

*QTc* - Since the QT varies according to the HR, it is usually corrected (QTc) by the Bazett's formula, where:



* QT measured in milliseconds and RR distance measured in seconds.

***Variants of ventricular repolarization: e arly repolarization pattern*** - Characterized by a J-point elevation ≥ 1 mm, leading to a lack of coincidence between the QRS and the baseline, generating an ST segment with an upper concavity in at least two contiguous precordial leads, with values also ≥ 1 mm.^7-10^

### Analysis of supraventricular arrhythmias

#### Supraventricular Arrhythmias

##### Presence of sinus P wave

*Sinus arrest(SA)* - A pause in sinus activity > 1.5 times the basic PP cycle.

*Second-degree sinoatrial block* - Leads to a lack of registration of the P wave in a cycle. Type I sinoatrial block (SAB I) is characterized by PP cycles progressively shorter until the blockade occurs. Type II sinoatrial block (SAB II) shows no difference between the PP cycles and the pause corresponds to two previous PP cycles. First-degree sinoatrial blocks are not visible on standard ECG. Third-degree blocks are observed as an atrial or junctional escape rhythm.

*Interatrial blocks* - Conduction delay between the right and left atria, which can be of first degree (P-wave duration ≥ 120 ms), third degree or advanced (P-wave duration ≥ 120 ms, biphasic or with a plus-minus morphology in the inferior wall, related to supraventricular arrhythmias, Bayés syndrome), and second degree, when these patterns appear transiently.^11^

*Pauses* - Tracing pauses may be related to the occurrence of sinus arrest, nonconducted atrial extrasystole, sinoatrial block, and AV block.

##### Occurrence of a non-sinus P wave before the QRS complex

*Ectopic atrial rhythm -* Originates in the atrium but in a different location than that of the anatomic region of the sinus node.

*Multifocal atrial rhythm -* Originates in multiple atrial foci, with an HR < 100 bpm, recognized in the ECG by the presence of ≥ 3 P-wave morphologies.

*Atrial escape beat* - Beat of atrial origin consequent to a temporary inhibition of the sinus node, generated to compensate for the absence of sinus activity.

*Atrial extrasystole(AE)* - Early atrial ectopic beat.

*Blocked atrial extrasystole* - Ectopic beat originating in the atrium which cannot be conducted to the ventricle; therefore, a QRS complex is not generated.

*Atrial tachycardia* - Atrial rhythm originating in a region other than the sinus node, characterized by the occurrence of a different P wave than that of the sinus, with an atrial rate > 100 bpm.

*Multifocal atrial tachycardia* - Presents the same characteristics as those of multifocal atrial rhythm, with an atrial rate > 100 bpm.

##### Absence of an antegrade P wave

*Atrial fibrillation(AF)* - Disorganized atrial electrical activity, with an atrial rate between 450 and 700 cycles/min and a variable ventricular response. The baseline can be isoelectric, with fine or coarse irregularities, or a combination of these changes ("f" waves).

*Atrial flutter* - Organized atrial electrical activity; the most common type has a counterclockwise direction with frequencies between 240 and 340 bpm (Type I) and a characteristic "F" wave pattern with a sawtooth pattern, which is negative in the inferior leads and generally positive in V1.

*Junctional rhythm* - Supplementary or replacement rhythm originating in the AV junction, with a similar morphology and duration of the baseline rhythm.

*Junctional extrasystole* - Early ectopic beat originating in the AV junction.

*Typical AV nodal reentrant tachycardia (AVNRT)* -^12^ Arises in the AV node secondary to nodal reentry, and the circuit uses a fast retrograde pathway and a slow antegrade pathway. If the baseline QRS is normal and narrow, we may observe pseudo "s" waves in the inferior wall and an rSr' (pseudo r') morphology in V1 during tachycardia.

*Atypical AV nodal reentrant tachycardia* - The origin location and the circuit are similar to those of typical AVNRT, but the direction of the activation is reversed.

*Orthodromic atrioventricular reentrant tachycardia* - Uses the normal conduction system in the anterograde direction and an accessory pathway in the retrograde direction. The QRS is generally narrow, and the P wave is retrograde, usually located in the ST segment.

##### Supraventricular arrhythmias with a wide QRS complex

*Aberrant conduction* - Supraventricular stimulus with compromised regional propagation in the conduction system, generating a QRS complex with a bundle-branch block morphology.

*Atrial extrasystole with aberrant conduction* - Atrial beat with a P wave followed by a QRS with a bundle-branch block morphology.

*Supraventricular tachycardia with aberrant conduction* - Generic denomination for the aforementioned tachycardias presenting with aberrant conduction.

*Antidromic atrioventricular reentrant tachycardia* - Uses an accessory pathway in an anterograde direction and the conduction system in a retrograde direction; aberrant QRS; is characterized by the morphologic pattern of preexcitation evident in QRS complexes.

*Criteria to differentiate wide QRS complex tachycardias* -(13 )The diagnosis of ventricular tachycardia (VT) includes the presence of an AV dissociation, with greater ventricular than atrial rate, or the presence of fusion and capture beats, as well as the presence of tachycardia with wide QRS in the occurrence of a prior acute myocardial infarction. When these signs are absent, algorithms such as those by Brugada and Vereckei, are available to help differentiate these tachycardias.^14,15^

## Analysis of ventricular arrhythmias

### Ventricular Arrhythmias

***Ventricular parasystole*** - Beat that originates in a ventricular focus and competes with the physiological heart rhythm (a parallel pacemaker with a permanent entry block and an occasional exit block).

***Idioventricular escape rhythm*** - Rhythm that originates in the ventricle, with an HR < 40 bpm, replacing anatomically higher rhythms that are temporarily inhibited.

***Ventricular escape beat(s)*** - Beat(s) originating in the ventricle as a consequence of temporary inhibition of anatomically higher rhythms; occur late, since they are subsidiary.

***Idioventricular accelerated rhythm (IVAR)*** - Originates in the ventricle (wide QRS), with an HR > 40 bpm, as a result of increased automaticity.

***Ventricular extrasystole (VE)*** -( 16) Beat originating early in the ventricle, with a postextrasystole pause when the RR interval recycles.

***Fusion beat*** - Originates in the ventricle and merges with beats from the physiological cardiac rhythm.

***Conducted supraventricular beat(s) during ventricular rhythm*** - Originates in the atrium, manages to overcome the (anatomical or functional) conduction block in the AV junction and partially or entirely depolarize the ventricle.

***Sustained monomorphic ventricular tachycardia (SMVT)*** - Ventricular rhythm with at least three successive beats, uniform morphology, and a rate > 100 bpm.

***Polymorphic ventricular tachycardia*** - Ventricular rhythm with a QRS of variable morphology and a rate > 100 bpm.

***Torsades de Pointes ventricular tachycardia (TdP)***- Wide QRS polymorphic tachycardia, generally self-limited, with a QRS that "rotates" around the baseline.

***Bidirectional tachycardia*** -^17^ Tachycardia of ventricular origin where the right branch (or rarely the left branch) is constantly blocked while conducting across the ventricle, with alternating blockade of the anterosuperior and posteroinferior divisions of the left branch, beat by beat.

***Ventricular fibrillation (VF)***- Characterized by bizarre and chaotic waves, with variable amplitude and frequency. This rhythm may be preceded by VT or TdP that degenerated into VF.

## Atrioventricular conduction

**Normal atrioventricular relations** - The period from the beginning of the P wave to the beginning of the QRS complex determines the PR interval, a moment when the atrial activation and the physiological retardation in the AV junction occur, with a duration of 120 ms to 200 ms.

**Delayed atrioventricular conduction** -^18,19^ Occurs when the atrial impulses experience a delay or fail to reach the ventricles.

***First-degree atrioventricular block*** - PR interval > 200 ms in adults with an HR between 60 and 90 bpm.

***Type I second-degree atrioventricular block (Mobitz type I)*** - The AV conduction gradually slows down in this condition *(Wenckebach phenomenon)*. There is a progressive increase in the PR interval, with gradually shorter increases, until the AV conduction is blocked and the atrial beat is unable to conduct.

***Type II second-degree atrioventricular block (Mobitz type II)*** - Sudden failure in the AV conduction. It is observed as a 1:1 AV conduction with a fixed PR interval and a sudden P-wave block.

***2:1 atrioventricular block*** - For every two beats originating in the atrium, one is conducted and depolarizes the ventricle, while the other is blocked and unable to depolarize it.

***Advanced or high-degree atrioventricular block*** - The AV conduction occurs in less than half of the atrial beats, in a proportion of 3:1, 4:1, or greater.

***Third-degree or total atrioventricular block (TAVB)*** - Stimuli originating in the atrium are unable to reach and depolarize the ventricles; thus, a focus below the blocked region takes over the ventricular rhythm. As a result, there is no correlation between atrial and ventricular electrical activity rates, which translates as P waves unrelated to QRS complexes. The frequency of the atrial rhythm is greater than that of the escape rhythm.

***Paroxysmal atrioventricular block*** - Sudden and unexpected occurrence of a succession of blocked P waves.

### Preexcitation

***Ventricular preexcitation*** - Characteristics of the classic pattern include a PR interval < 120 ms during sinus rhythm in adults and < 90 ms in children; a notch in the initial portion of the QRS complex (delta wave) interrupting the P wave or appearing immediately after its end; QRS duration > 120 ms in adults and > 90 ms in children; secondary changes in ST and T.

### Other Mechanisms of Changes in the Normal AV Relationship

***Atrioventricular dissociation*** - Occurrence of two dissociated rhythms, one of atrial origin (usually a sinus rhythm with a regular PP) and the other of junctional or ventricular origin. The frequency at both foci may be similar (isorhythmic dissociation).^20^

***Retrograde atrial activation*** - The atrium is activated by a ventricular stimulation that is conducted retrogradely, usually through the AV node or an anomalous track. A wide QRS (of ventricular origin) is observed, followed by a negative P wave in the inferior leads.

## Overload of the cardiac chambers

### Atrial Overloads

***Left atrial overload (LAO)*** - Increase in P wave duration ≥ 120 ms, associated with the emergence of a notch in D2, and a P wave with an increased negative component in the V1 lead (Morris index).

***Right atrial overload (RAO)*** - P wave with peak and amplitude greater than 0.25 mV or 2.5 mm and, in the V1 lead, a positive initial portion > 0.15 mV or 1.5 mm.

***Biatrial overload (BAO)*** - An association of the LAO and RAO criteria.

#### Ventricular overloads

**Left ventricular overload (LVO)** ^21-23^

*Romhilt-Estes criteria* -^24^ According to these criteria, an LVO occurs when five or more points are reached in the following scores:

*Three-point* c*riteria* - Increased QRS amplitude; a strain pattern of repolarization in the absence of digitalis influence; and the Morris index.

*Two-point criteria* - Deviation of the QRS electrical axis beyond -30º.

*One-point criteria* - Increase in the ventricular activation time (VAT) or intrinsicoid deflection greater than 40 ms; increased QRS duration (> 90 ms) in V5 and V6; and a strain pattern under digitalis influence.

*Sokolow-Lyon index* -^22^ Considered positive when the sum of the amplitude of the S wave in the V1 lead with the amplitude of the R wave in the V5/V6 leads is > 35 mm.

*Cornell index -* When the sum of the amplitude of the R wave in the aVL lead with the amplitude of the S wave in V3 is > 28 mm in men and > 20 mm in women.

*Changes in ventricular repolarization -* Flat T wave in the left leads (D1, aVL, V5, and V6) or a strain pattern (ST depression with negative and asymmetric T wave).

#### Right ventricular overload (RVO) ^25^

*Axis -* Electrical axis of the QRS complex in the frontal plane located to the right of +110(o) in adults.

*Wide R wave -* Presence of a high-voltage R wave in V1 and V2, and deep S waves in V5 and V6.

*qR or qRs morphology -* A qR or qRs morphology in V1, or V1 and V2, is one of the most specific signs of RVO.

*rsR' morphology -* A triphasic pattern (rsR'), with a more prominent R' wave in the right precordial leads V1 and V2.

*T wave -* Positive T waves in V1 after 3 days of life and up to the age of 6 years, when the R/S ratio in this lead is greater than 1.

*Ventricular repolarization -* A strain pattern of repolarization in the right precordial leads.

*Index* - A > 10.5 mm sum of R in V1 + S in V5-V6.

#### Biventricular overload

- QRS electrical axis in the frontal plane deviated to the right, associated with voltage criteria for LVO.
- ECG typical of RVO associated with one or more of the following elements:
  b.1) deep Q waves in V5 and V6, and in the inferior leads.
  b.2) R voltage increased in V5 and V6.
  b.3) S in V1 and V2 + R in V5 and V6 with positive Sokolow criteria.
  b.4) intrinsicoid deflection in V6 ≥ 40 ms.
- Wide, isodiphasic, R/S type QRS complexes in intermediate precordial leads from V2 to V4.

## Analysis of intraventricular blocks (delay, conduction delay)

**Intraventricular Blocks **-^26^ Changes in the intraventricular propagation of electrical impulses, changing the form and duration of the QRS complex, which can be frequency-dependent and fixed or intermittent.

### Left Bundle-Branch Block^27,28^

- wide QRS with a duration ≥ 120 ms as an essential condition.
- absence of "q" in D1, aVL, V5, and V6; variants may have a "q" wave in aVL only.
- wide R waves with notches and/or mid-terminal slurring in D1, aVL, V5, and V6.
- "r" wave with slow growth from V1 to V3, with possible occurrence of QS.
- wide S waves with thickening and/or notches in V1 and V2.
- intrinsicoid deflection in V5 and V6 ≥ 50 ms.
- QRS electrical axis between -30° and +60°.
- ST depression and asymmetrical T in opposition to the mid-terminal delay.

### Left bundle-branch block in association with LVO^29^

- left atrial enlargement.
- QRS duration > 150 ms.
- R wave in aVL > 11 mm.
- S waves > 30 mm in V2 and > 25 mm in V3.
- SâQRS beyond - 40°.
- presence of a Sokolow-Lyon index ≥ 35 mm.

### Left bundle-branch block in association with RVO

- low voltage in the precordial leads.
- prominent R wave in aVR.
- R/S ratio lower than 1 in V5.

### Right Bundle-Branch Block

- wide QRS with a duration ≥ 120 ms as an essential condition.
- slurred S waves in D1, aVL, V5, and V6.
- qR waves with slurred R in aVR.
- rSR' or rsR' with thickened R' in V1.
- variable QRS electrical axis, tending to the right in the frontal plane.
- asymmetrical T wave opposed to a delay at the end of the QRS.

***End conduction delay - *** This expression may be used when the conduction disturbance in the right branch is very subtle.

### Left fascicular blocks^30,31^

**Left anterosuperior fascicular block (LAFB) ^32^**

- QRS electrical axis ≥ -45°.
- rS in D2, D3, and aVF with an S3 greater than S2; QRS duration < 120 ms.
- S wave in D3 with an amplitude greater than 15 mm (or an equivalent area).
- qR in D1 and aVL with an intrinsicoid deflection time greater than 50 ms or qRs with a minimal "s" in D1.
- qR with slurred R in aVL.
- slow r wave progression from V1 to V3.
- presence of S from V4 to V6.

#### Left anteromedial fascicular block (LAFB) ^33,34^

- R wave ≥ 15 mm in V2 and V3 or starting in V1, increasing towards the intermediary precordial leads and decreasing from V5 to V6.
- "r" wave with sudden growth jump from V1 to V2 ("rS" in V1 to R in V2).
- QRS duration < 120 ms.
- absence of a QRS electrical axis deviation in the frontal plane.
- T waves generally negative in the right precordial leads.
- qR morphology in V1 to V4.

#### Left posteroinferior fascicular block (LPIFB)

- QRS electrical axis in the frontal plane oriented to the right > +90°.
- qR in D2, D3, and aVF with R3 > R2 and an intrinsicoid deflection > 50 ms.
- R wave in D3 > 15 mm.
- intrinsicoid deflection duration increased in aVF, V5-V6 greater than or equal to 50 ms.
- rS in D1 with a duration < 120 ms; slower progression of "r" may occur from V1-V3.
- S wave from V2 to V6.

### Right fascicular blocks

**Right superior fascicular block**
- rS in D2, D3, and aVF with an S2 > S3.
- Rs in D1 with an s wave > 2 mm, rS in D1 or D1, D2 and D3 (S1, S2, S3) with a duration < 120 ms.
- slurred S in V1 - V2/V5 - V6 or, eventually, rSr' in V1 and V2.
- qR with a slurred R in avR.

**Right inferior fascicular block**
- R wave in D2 > R wave in D3.
- rS in D1 with a duration < 120 ms.
- QRS electrical axis in the frontal plane oriented to the right > +90°.
- slurred S in V1-V2/V5-V6 or, eventually, rSr' in V1 and V2.
- qR with slurred R in aVR.

### Special situations involving intraventricular conduction

***Peri-infarction block*** - ^35^ Increased duration of the QRS complex in the presence of an abnormal Q wave due to myocardial infarction in the inferior or lateral leads, with an increased final portion of the QRS complex directed opposite to the Q wave (*i.e.,* QR complex).

***Peri-ischemia block*** - ^35^ Transient increase in the duration of the QRS complex accompanied by an ST-segment deviation seen in the acute phase.

***Fragmentation of the QRS complex (fQRS)*** - ^36^ Presence of notches in the R or S wave in two contiguous leads in the absence of bundle-branch block or, when the block is present, the finding of more than two notches.

***Atypical left bundle-branch block - ***^37^ Occurrence of infarction in a patient with a prior left bundle-branch block. In this condition, we have the presence of wide and deep Q waves, QS pattern in V1-V4, and QR in V5-V6, with QRS fragmentation.

***Parietal or Purkinje/muscle intraventricular block - ***^38^ When the dromotropic disturbance is located between the Purkinje fibers and the muscle, observed in severe hypertrophy and cardiomyopathies.

## Analysis of electrically inactive areas

**Definition of an electrically inactive area (EIA)** - An EIA is considered when the ventricular activation does not occur as expected and does not suggest an intraventricular conduction disturbance. It is characterized by the presence of pathological Q waves in two contiguous leads.

### Topographic analysis of ischemic manifestations

**Topographic analysis of ischemic manifestations on the ECG**

- anteroseptal wall - V1, V2, and V3 leads.
- anterior wall - V1, V2, V3, and V4 leads.
- localized anterior wall - V3, V4 or V3-V5 leads.
- anterolateral wall - V4 to V5, V6, D1, and aVL leads.
- extensive anterior wall - V1 to V6, D1, and aVL.
- low lateral wall - V5 and V6 leads.
- high lateral wall - D1 and aVL.
- inferior wall - D2, D3, and aVF.

The terms "posterior wall" and "dorsal wall" should no longer be used.^39^

### Infarctions in special locations

***Right ventricular myocardial infarction - ***ST-segment elevation in right precordial leads, particularly with an elevation of the ST segment above > 1 mm in V4R.

**Atrial infarction - **Visible in the presence of > 0.5 mm PR segment elevation or depression.

### Diagnostic criteria for myocardial ischemia

**Presence of ischemia**

- Subendocardial ischemia: presence of a positive, symmetric, and peaked T wave.
- Subepicardial ischemia: presence of a negative, symmetric, and peaked T wave; this finding is currently attributed to a reperfusion pattern.

***Secondary changes in the T wave*** - are those not fitting within the definition of ischemic waves.

### Diagnostic criteria for the presence of a lesion

Subepicardial lesion: J point and ST-segment elevation, with a superior concavity or convexity (more specific) in two contiguous leads that explore the involved region, of at least 1 mm in the frontal plane and left precordial leads. For the precordial leads V1 to V3, consider ST-elevations as ≥ 1.5 mm in women, ≥ 2.0 mm in men older than 40 years, and ≥ 2.5 mm in men younger than 40 years.^40^

Subendocardial lesion: ^40^ depression of the J point and ST segment, horizontal or down-sloping ≥ 0.5 mm in two contiguous leads that explore the regions involved, measured 60 ms after the J point.

### Differential diagnoses

***Subepicardial ischemia - *** Must be distinguished from secondary alterations in ventricular repolarization in LVO or bundle-branch blocks.

***Acute myocardial infarction with ST elevation -*** Must be distinguished from the following situations:

- Early repolarization.
- Pericarditis and myocarditis.
- Prior acute myocardial infarction with a dyskinetic area and persistent elevation.
- Acute abdominal conditions.
- Hyperkalemia.
- Catecholaminergic syndromes.

### Association of myocardial infarction with bundle-branch blocks

***Myocardial infarction in the presence of right bundle-branch block (RBBB) - *** The presence of an RBBB usually does not prevent the recognition of an associated myocardial infarction.

***Myocardial infarction in the presence of left bundle-branch block (LBBB) - ***The presence of an LBBB hinders the recognition of an associated myocardial infarction. The occurrence of ST-segment elevation or depression may allow identification of a recent myocardial infarction, according to the criteria defined by Sgarbossa et al.:^41^

- ST-segment elevation ≥ 1.0 mm in agreement with the QRS/T.
- ST-segment depression ≥ 1.0 mm in V1, V2, and V3.
- ST-segment elevation ≥ 5.0 mm in disagreement with the QRS/T.

**Rules on clinical suspicion of acute ischemic disease - **A normal ECG does not rule out the presence of coronary heart disease, and the recommendations of specific guidelines for acute coronary syndromes must be followed.^42,43^

## Artificial cardiac pacing

**Artificial cardiac pacing (ACP) - **The recognition of an impaired antibradycardia function is feasible and necessary.

The standardization of the terms used to describe the operations of the ACP systems follows an international standardization developed by the *North American Society of Pacing and Electrophysiology* (NASPE) and the *British Pacing and Electrophysiology Group* (BPEG). Therefore, some of the established terms are used in English.

### Five letter code

First letter: chamber paced

O: none

A: atrium

V: ventricle

D: atrium and ventricle

Second letter: chamber sensed

O: none

A: atrium

V: ventricle

D: atrium and ventricle

Third letter: response to sensing

O: none

I: inhibited

T: triggered

D: inhibited and triggered

Fourth letter: rate modulation

O: none

R: rate response sensor enabled

Fifth letter: multisite pacing

O: none

A: atrium

V: ventricle

D: atrium and ventricle

### Basic terminology

- spike: a graphic representation of the electrical stimulation produced by the ACP system.
- capture: artificial tissue depolarization (caused by the occurrence of the spike).
- basic rate: frequency in which the pacemaker stimulates the heart (atrium and/or ventricle) without the interference of spontaneous beats.
- atrioventricular interval (AVI): interval between a spontaneous atrial activity (perceived) or stimulated and the ventricular stimulation.
- hysteresis: time interval above the basic rate, triggered by a spontaneous event.
- maximum rate (MR): maximum stimulation rate.
- sensitivity: ability to recognize atrial or ventricular spontaneous electrical events.
- normal inhibition: absence of spike emission by the pulse generator when the atrial or ventricular channel "feels," respectively, a P wave or a spontaneous QRS.

### Analysis of the electrocardiographic characteristics of ACP systems

- normofunctioning ACP system: normal capture and sensitivity.
- ACP system with a minimal role of ventricular stimulation: in AV systems, there is an increase in the AVI or periodic change to the AAI operating mode, with the aim of seeking a spontaneous AV conduction.
- loss of atrial and/or ventricular capture (intermittent or persistent): inability of a spike to trigger depolarization of the paced chamber.
- sensitivity failure:
  d.1) excessive sensitivity (oversensing): exaggerated sensitivity, resulting in misidentification of an electrical signal that does not correspond to the depolarization of the related chamber (electromagnetic interference, myopotentials, T wave, etc.).
  d.2) decreased sensitivity (undersensing): inability to recognize spontaneous depolarization. It may occur due to inadequate planning or modifications in the capture of intrinsic signal (in which the system does not "see" the P wave or the QRS complex).
- fusion beats: correspond to the artificial activation of the cardiac tissue simultaneously to the spontaneous depolarization, causing complex hybrids.
- pseudofusion beats: spontaneous activation of the cardiac tissue simultaneous to the emission of the spike of the pacemaker, which has no effect on the QRS complex or P wave (ventricular and atrial pseudofusion, respectively).
- pacemaker-mediated tachycardia: arrhythmia restricted to the AV stimulation systems, characterized by ventricular triggering from a retrograde P wave.
- pacemaker-conducted tachycardia: tachyarrhythmia that involves the AV stimulation systems, characterized by the presence of supraventricular arrhythmia that when perceived by the atrial channel triggers ventricular captures in higher frequencies, maintaining certain characteristics of spontaneous arrhythmia.
- pacemaker-induced tachycardia: changes in sensitivity or electromagnetic interference causing arrhythmias.

## Criteria for characterization of pediatric electrocardiograms

**Analysis of pediatric electrocardiograms -**^44,45^ Difficulties in establishing normal electrocardiographic standards in children arise from various aspects that should always be considered in the analysis of pediatric ECGs:

- The characteristics of the electrocardiographic tracing should be assessed according to the age of the child.
- The existence of a thoracic deformity or poor cardiac position limits the interpretation of the ECG.
- The ECG of newborns reflects the hemodynamic repercussions on the right ventricle occurring during the intrauterine life and the anatomic and physiological changes deriving from the transition of the fetal circulation to the neonatal circulation.
- The ECG in children shows the progressive reduction in the right ventricle domain until it reaches the characteristic pattern of physiological left ventricular predominance, as observed in the ECG in adults.

Table 1, elaborated by Davignon et al.,^44^ presents the reference values of the electrocardiographic parameters in children at various ages. It shows the main ECG changes in the first year of life, particularly in the neonatal period (from day 1 to 30).

### Special considerations in the analysis of pediatric electrocardiograms

***Definition of* situs** - It is based on the location of the sinus node. In *situssolitus*, the P wave axis is around +60°, while in *situs inversus*, the P axis is +120°, with a consequently negative P wave in D1.

***"q" waves*** - The presence of a "q" wave in V1 is always considered pathological, while in V6, it is present in 90% of the children above the age of 1 month.

***T wave*** - It may be negative in D1 and positive in aVR in the first hours of life. In the first 48 hours of life, the T wave tends to be positive in V1, becoming negative after 3 to 7 days and becoming then again positive in pre-adolescence.

## The ECG in channelopathies and other genetic alterations

**Genetics and the electrocardiogram - **An innovative field in cardiology that emerged in recent years was the report of potentially fatal clinical conditions that present a characteristic electrocardiographic pattern, and the improvement in genetic mapping techniques.

**Channelopathies** - Cardiac channelopathies result from genetic mutations or acquired malfunctioning of ion channels responsible for triggering changes in depolarization or repolarization in the action potential phases of the cell.

**Congenital long QT syndrome **-^46^ Its main characteristic is the prolongation of the QTc interval on the ECG, with values > 460 ms. Clinically, the presence of syncope or cardiac and respiratory arrest triggered by emotional and physical stress should raise the hypothesis of long QT (LQT) syndrome. ECG characteristics:

- LQT1: normal T wave with a wide base and late start.
- LQT2: T wave with low amplitude.
- LQT3: late T wave, after a long and straight ST.

**Short QT syndrome **-^47^ Recently described entity (2000) characterized by the finding of a short QT interval associated with AF and sudden cardiac death. The genetic defect in this condition is an increased functioning of potassium channels, which operate in the third phase of the action potential, shortening the QT interval. The syndrome is mainly related to the *KCNH2*, *KCNQ1*, and *KCNJ2* genes. ECG findings include a short QTc interval (< 370 ms) and a distance between the J point and the peak of the T wave < 120 ms.

**Brugada syndrome** -^48,49^ Channelopathy expressed by a defect in sodium channels in the right ventricle (RV) epicardium. This syndrome affects mainly men and individuals with a family history of sudden death. It is transmitted by autosomal dominant inheritance and is responsible for 20% of the sudden deaths with a normal heart at autopsy. It is genetically heterogeneous, involving at least 13 genes. On the ECG, it is characterized by a J-point elevation in the V1 and V2 leads, with two possible presentations:

- Type 1 pattern: coved ST-segment elevation ≥ 2 mm, followed by T-wave inversion, with a significantly slow R' decline.
- Type 2 pattern: saddle-shaped ST elevation with a ≥ 2 mm peak and ≥ 1 mm base.

**Catecholaminergic tachycardia **-^50^ The following characteristics should raise suspicion of this condition: history of sudden death in the family with reports of syncope starting during childhood and adolescence, associated with ventricular extrasystoles with an HR between 110 and 130 bpm (generally isolated, intermittent, bigeminal and paired), which are usually interrupted by an increased HR. Approximately 30% of the individuals may present QT intervals between 460 ms and 480 ms. Between 50 to 60% of the cases of catecholaminergic polymorphic VT (PVT) derive from inherited or sporadic mutations in ryanodine channels, which are responsible for regulating the intracellular calcium.

## Genetic diseases with primary cardiac involvement

***Arrhythmogenic right ventricular dysplasia***-^51^ Genetic disease with primary involvement of the RV (replacement of myocytes with fibrofatty tissue), associated with arrhythmias, heart failure, and sudden death. The ECG is characterized by the presence of a delay in the conduction of the QRS, with a low voltage and longer duration (epsilon wave, present in 30% of the cases), associated with rounded and asymmetrical negative T waves in V1 to V4 leads. Association with extrasystoles originating in the RV.

***Hypertrophic cardiomyopathy*** - ^52^ A primarily cardiac disease of genetic basis with autosomal dominant inheritance, with 23 described genetic mutations. It occurs with severe segmental or diffuse left ventricular hypertrophy. The ECG is altered in at least 75% of the patients, with a good sensitivity for the pediatric age group. It is characterized by rapid and deep Q waves in inferior and/or precordial leads, generally associated with classical signs of LVO and accompanied by characteristic ST-T changes.

## Genetic diseases with secondary cardiac involvement

***Muscular dystrophy*** -^53^ The most common electrocardiographic findings are the presence of a wide R wave (ratio R/S > 1) in V1 and V2; deep Q wave in V6, DI, and aVL; right bundle-branch conduction delay; QS complexes in I, aVL, D1, D2, and D3; and ventricular repolarization changes.

## Electrocardiographic alterations in athletes

**The importance of the electrocardiogram of athletes -** ^54,55^ It is currently necessary to understand the "athlete's ECG," *i.e.*, electrocardiographic changes arising from high-performance training, without a necessary presence of anatomical and/or structural changes, considered as part of the "athlete's heart."

### Common electrocardiographic changes or related to training:

- sinus bradycardia.
- sinus phasic or respiratory arrhythmia.
- increased PR interval.
- increased voltage of the R or S waves between 30 to 35 mm.
- change in ventricular repolarization, of the early repolarization type.
- right bundle-branch conduction delay.
- second-degree AV block, Mobitz I type.
- ventricular overload pattern by voltage criteria only (Sokolow-Lyon).

## Characterization of special clinical/systemic situations

### Clinical conditions that alter the electrocardiogram

***Digitalis action*** - ST-T depression with superior concavity ("spooned" T wave); decreased QTc interval. In digitalis intoxication, several arrhythmias may occur, predominantly ventricular extrasystole.

***Drug-induced ST-T changes*** - Increased QTc interval.

***Electrical* alternans** - Presence of QRS with alternately higher and lower amplitudes in successive QRS complexes.

***T-wave* alternans - **Characterized by a variation in T-wave amplitude, shape, and orientation, beat by beat. These variations may be episodic or permanent.

***Atrial septal defect*** - Delay in the end conduction through the right branch and possible association with RVO.

***Pericardial effusion*** - Low QRS voltage, sinus tachycardia, and electrical *alternans*.

***Dextrocardia with* situs inversus totalis** - P wave negative in D1 and positive in aVR; negative QRS complexes in D1 and aVL, and progressively lower from V1 to V6.

***Dextroposition*** - May manifest with a negative or plus-minus P wave in D1, deep Q wave in D1 and aVL, and qRS complexes in right precordial leads.

#### Electrolyte disorders

*Hyperkalemia* - The changes depend on the serum potassium concentrations; occur in a sequence: T wave with increased amplitude, symmetric, and with a narrow base; decreased QTc interval; intraventricular conduction disturbance; decreased P wave amplitude until disappearance, with the presence of sinoventricular conduction.

*Hypokalemia* - Increased U wave amplitude; ST segment and T-wave depression; increased QTU interval.

*Hypocalcemia* - Straightening and increased duration of the ST segment with a consequent increase in the QTc interval.

*Hypercalcemia* - Shortening and eventual disappearance of the ST segment.

***Chronic obstructive pulmonary disease*** - Rightward shift of the P wave axis (close to +90(o)); low QRS voltage; rightward shift of the QRS axis; posterior deviation of the transition precordial QRS zone to the left (rS from V1 to V6).

***Low QRS voltage*** - Low voltage of the QRS complex across the tracing (< 0.5 mV in the frontal plane leads and < 1.0 mV in the precordial leads).

***Pulmonary embolism***- Sinus tachycardia, end conduction delay in the right branch, acute deviation of the QRS axis to the right, and negative T waves in the anterior wall of the LV.

***Ashman (or Gouaux-Ashman) phenomenon***- Aberrant conduction that occurs in a beat that immediately follows a short cycle after a long cycle, due to an increased refractory period in the conduction system, found more frequently in AF.

***Hypothermia***- Bradycardia, presence of J or Osborn wave.

***Hypothyroidism***- Bradycardia and low QRS voltage.

***Chronic renal failure***- Association of the hyperkalemia and hypocalcemia alterations.

***Acute impairment of the central nervous system***- Giant negative T waves simulating subepicardial ischemia (cerebral T wave); increased QTc interval.

***Pericarditis - *** The inflammatory process arising from underlying epicarditis in the ventricles is responsible for the following electrocardiographic changes:

- T wave: slightly increased and symmetrical in the initial phase. Characteristically, it is not inverted in the presence of manifestations of ST elevation.
- ST segment: diffuse elevation with upper concavity.
- PR segment depression.

## Criteria for technical evaluation of the tracings

**Calibration of the electrocardiograph - **In modern computerized equipment with digitized tracings, the calibrator pattern is verified automatically. The normal pattern must have 1 mV (10 mm).

### Positioning of the electrodes

***Swapped limb electrodes - *** Show D1 leads with negative waves and aVR with positive waves.

***Right lower limb electrode swapped with one from the superior limbs - *** Small wave amplitudes in D2 (right arm) or D3 (left arm).

***Swapped precordial electrodes - ***Change in the normal progression of the R wave from V1 to V6.

***Misplacement of the V1 and V2 electrodes - ***V1 and V2 electrodes positioned incorrectly above the second intercostal space may produce an rSr' pattern simulating an end conduction delay, or an rS morphology from V1 to V3, and negative P wave in V1, simulating an LAO.

***Alterations resulting from improper operation of software and systems for the acquisition of computerized electrocardiographic signs - ***The use of data acquisition by computerized systems has begun to reveal specific new problems that are not yet fully known.

### Other Interferences

***Muscle tremors*** - Muscle tremors can interfere with the baseline, mimicking electrocardiographic changes such as AF and VF in patients with Parkinson's disease.

***Neurostimulation*** - Patients with disorders of the central nervous system requiring the use of artificial electrical stimulation devices may present artifacts that mimic the spike of a cardiac pacemaker.

***Cold, fever, hiccups, and psychomotor agitation -*** Also produce artifacts in the baseline.

***Large precordial electrode - *** The application of the conductive gel in a continuous strip in the precordium results in similar tracings from V1 to V6, corresponding to the average of the electrical potentials in these leads.^2^

**Automated reports** - Metric and vectorial measurements are not recommended, neither reports obtained with these systems, since the report is a medical act.

**Reports via the *internet - *** Tele-ECG^56^ systems register electrocardiographic tracings obtained remotely by using different means and data transfer technologies able to accurately reproduce the examination conducted with 12 simultaneous leads following national and international guidelines.

## Footnotes

* (para referências completas, consultar "Pastore CA, Pinho JA, Pinho C, Samesima N, Pereira-Filho HG, Kruse JCL, et al. III Diretrizes da Sociedade Brasileira de Cardiologia sobre Análise e Emissão de Laudos Eletrocardiográficos. Arq Bras Cardiol 2016; 106(4Supl.1):1-23"
